# Supplementary material for: Touching Emotions: How Touch Shapes Facial Emotional Processing Among Adolescents and Young Adults
Source: Int J Environ Res Public Health. 2025 Jul 15;22(7):1112. doi: 10.3390/ijerph22071112 (PMC12295908; doi:10.3390/ijerph22071112)
Supplement: Supplementary file 1 [file ijerph-22-01112-s001.zip › ijerph-3667037-supplementary.pdf]

# Supplementary Materials

*Touching emotions: how touch shapes facial emotional processing among adolescents and young adults*

## Pre-test subjective pleasantness of touch

| Tested models | Variables | AIC | ΔAIC | wAIC | $R^2$ | χ2 | *p* |
|---|---|---|---|---|---|---|---|
| Model 0 | Random effect of Participants | 1464.9 | | 0.00 | 0.024 | | |
| Model 1 | + Age group | 1466.8 | -1.94 | 0.00 | 0.025 | 0.06 | 0.799 |
| Model 2 | + Valence touch | 1276.9 | 189.9 | 0.34 | 0.461 | 191.9 | < 0.001 |
| Model 3 | + Age group × Valence touch | 1278.8 | -1.96 | 0.12 | 0.461 | 0.04 | 0.845 |
| Model 4 | + Social condition | 1280.7 | -1.83 | 0.05 | 0.461 | 0.17 | 0.682 |
| **Model 5** | **+ Age × Valence × Condition** | **1276.2** | **4.45** | **0.35** | **0.474** | **10.45** | **0.015** |
| Model 6 | + Gender | 1277.8 | -1.62 | 0.14 | 0.474 | 0.38 | 0.537 |

**Table S1.** Comparison between models predicting tactile preference scores. Note that smaller values of AIC indicate better fitting models.

MODEL INFO (Model 5):
Observations: 240
Dependent Variable: Preference score
Type: Mixed effects generalized linear regression
Error Distribution: poisson
Link function: log

MODEL FIT:
AIC = 1276.21, BIC = 1307.54
Pseudo-$R^2$ (fixed effects) = 0.47
Pseudo-$R^2$ (total) = 0.48

FIXED EFFECTS:

| | Est. | S.E. | z val. | p |
|---|---|---|---|---|
| (Intercept) | 2.27 | 0.06 | 38.37 | 0.00 |
| Age_Group (*reference category Adolescent*) | -0.07 | 0.09 | -0.84 | 0.40 |
| Valence (*ref Negative*) | 0.60 | 0.07 | 8.22 | 0.00 |
| Condition (*ref Non-social*) | 0.07 | 0.08 | 0.82 | 0.42 |
| Age_Group:Valence (*ref Adolescent - Negative*) | 0.01 | 0.10 | 0.12 | 0.90 |
| Age_Group:Condition (*ref Adolescent - Non-social*) | 0.10 | 0.12 | 0.86 | 0.39 |
| Valence:Condition (*ref Negative - Non-social*) | -0.22 | 0.10 | -2.09 | 0.04 |
| Age_Group:Valence:Condition (*ref Adolescent - Negative - Non-social*) | 0.01 | 0.15 | 0.10 | 0.92 |

ANOVA:

| | Chisq | Df | Pr(>Chisq) |
|---|---|---|---|
| (Intercept) | 1472.25 | 1 | < 0.001 *** |
| Age_Group | 0.70 | 1 | 0.403 |
| Valence | 67.53 | 1 | < 0.001 *** |
| Condition | 0.66 | 1 | 0.415 |
| Age_Group:Valence | 0.02 | 1 | 0.902 |
| Age_Group:Condition | 0.74 | 1 | 0.390 |
| Valence:Condition | 4.38 | 1 | 0.036 * |
| Age_Group:Valence:Condition | 0.01 | 1 | 0.924 |

POST-HOC CONTRASTS:

Condition = Non social:

| model term | df1 | df2 | F.ratio | p.value |
|---|---|---|---|---|
| Age_Group | 1 | Inf | 1.455 | 0.2278 |
| Valence | 1 | Inf | 33.40 | <.0001 |
| Age_Group:Valence | 1 | Inf | 0.015 | 0.9020 |

Condition = Social:

| model term | df1 | df2 | F.ratio | p.value |
|---|---|---|---|---|
| Age_Group | 1 | Inf | 0.634 | 0.4259 |
| Valence | 1 | Inf | 59.18 | <.0001 |
| Age_Group:Valence | 1 | Inf | 0.068 | 0.7938 |

**Table S2.** Description of the effects predicted by the best fitting model for preference score (Model 5), ANOVA and post-hoc contrasts

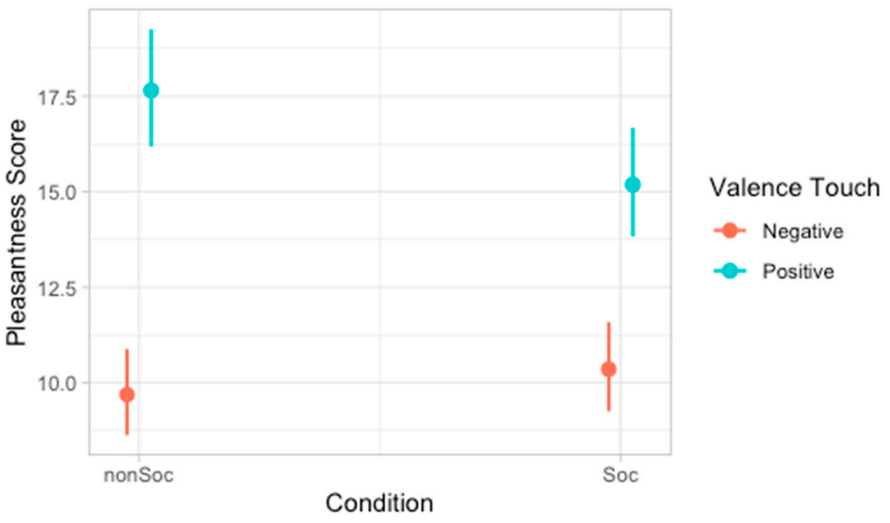


**Figure S1.** Plot of predicted values of preference score. Prediction lines and bars with confidence intervals (N participants = 60; N observations = 240).

**Experiment 1: Accuracy**

| **Tested models** | **Variables** | **AIC** | **ΔAIC** | **wAIC** | $R^2$ | **χ2** | ***p*** |
|---|---|---|---|---|---|---|---|
| Model 0 | Random effect of Participants | 550.0 | | 0.27 | 0.53 | | |
| Model 1 | + Age Group | 552.0 | -1.99 | 0.10 | 0.53 | 0.009 | 0.924 |
| Model 2 | + Face valence | 552.3 | -0.33 | 0.08 | 0.54 | 1.67 | 0.197 |
| Model 3 | × Face valence | 554.2 | -.1.84 | 0.03 | 0.54 | 0.16 | 0.693 |
| Model 4 | + Touch valence | 556.2 | -2.00 | 0.01 | 0.54 | 0.0003 | 0.986 |
| Model 5 | × Touch valence | 549.4 | 6.73 | 0.34 | 0.57 | 12.73 | 0.005 |
| Model 6 | + Condition | 550.8 | -1.40 | 0.17 | 0.57 | 0.60 | 0.437 |
| Model 7 | × Condition | 559.5 | -8.72 | 0.00 | 0.58 | 5.28 | 0.625 |
| Model 8 | + Gender | 561.5 | -1.98 | 0.00 | 0.57 | 0.02 | 0.892 |

**Table S3.** Comparison between models predicting accuracy scores. Note that smaller values of AIC indicate better fitting models.

MODEL INFO (Model 5):
Observations: 1907
Dependent Variable: ACC
Type: Mixed effects generalized linear regression
Error Distribution: binomial
Link function: logit

MODEL FIT:
AIC = 549.42, BIC = 599.40
Pseudo-$R^2$ (fixed effects) = 0.04
Pseudo-$R^2$ (total) = 0.57

FIXED EFFECTS:

| | Est. | S.E. | z val. | p |
|---|---|---|---|---|
| (Intercept) | 4.86 | 0.69 | 7.07 | 0.00 |
| Age Group (*reference category: Adolescent*) | -0.60 | 0.82 | -0.73 | 0.46 |
| Valence.Face (*ref Negative*) | -0.77 | 0.51 | -1.50 | 0.13 |
| Valence.Touch (*ref Negative*) | -1.06 | 0.50 | -2.10 | 0.04 |
| Age Group:Valence.Face (*ref Adolescent - Negative*) | 1.41 | 0.77 | 1.82 | 0.07 |
| Age Group:Valence.Touch (*ref Adolescent - Negative*) | 1.33 | 0.73 | 1.83 | 0.07 |
| Valence.Face:Valence.Touch (*ref Negative - Negative*) | 2.57 | 0.80 | 3.22 | 0.00 |
| Age Group:Valence.Face:Valence.Touch ) (*ref Adolescent - Negative - Negative)* | -3.37 | 1.12 | -3.00 | 0.00 |

ANOVA:

| | Chisq | Df | Pr(>Chisq) |
|---|---|---|---|
| (Intercept) | 49.9819 | 1 | < .001 *** |
| Age_Group | 0.5368 | 1 | 0.464 |
| Valence.Face | 2.2512 | 1 | 0.134 |
| Valence.Touch | 4.4193 | 1 | 0.036 * |
| Age_Group:Valence.Face | 3.3092 | 1 | 0.069 . |
| Age_Group:Valence.Touch | 3.3537 | 1 | 0.067 . |
| Valence.Face:Valence.Touch | 10.3621 | 1 | 0.001** |
| Age_Group:Valence.Face:Valence.Touch | 9.0074 | 1 | 0.003 ** |

POST-HOC CONTRASTS:

Age_Group = Adolescent:

| model term | df1 | df2 | F.ratio | p.value |
|---|---|---|---|---|
| Valence.Face | 1 | Inf | 1.826 | 0.1766 |
| Valence.Touch | 1 | Inf | 0.359 | 0.5491 |
| Valence.Face:Valence.Touch | 1 | Inf | 10.362 | 0.0013 |

Age_Group = Adult:

| model term | df1 | df2 | F.ratio | p.value |
|---|---|---|---|---|
| Valence.Face | 1 | Inf | 0.356 | 0.5507 |
| Valence.Touch | 1 | Inf | 0.101 | 0.7503 |
| Valence.Face:Valence.Touch | 1 | Inf | 1.032 | 0.3097 |

**Table S4.** Description of the effects predicted by the best fitting model for accuracy scores (Model 5), ANOVA and post-hoc contrasts

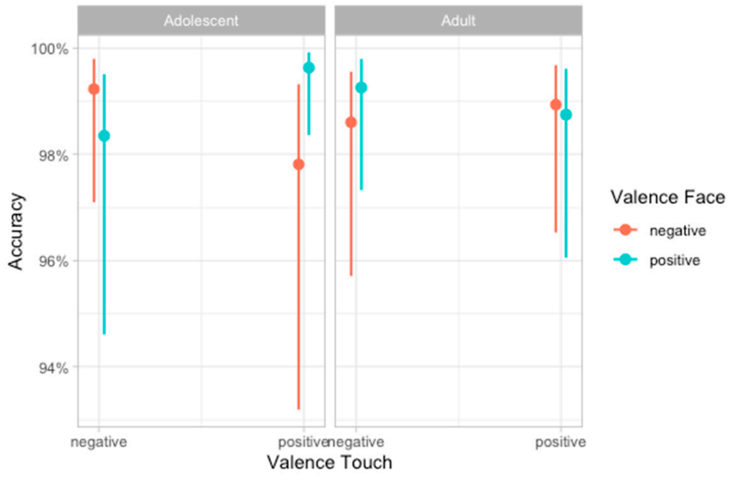


**Figure S2.** Plot of predicted values of accuracy showing the three-way-interaction effect between Age Group, Face valence and Touch valence. Prediction lines and bars with confidence intervals (N participants = 60; N observations = 1907)

**Experiment 1: RTs**

| Tested models | Variables | AIC | ΔAIC | wAIC | χ2 | *p* |
|---|---|---|---|---|---|---|
| Model 0 | Random effect of Participants | 986.6 | | 0.00 | | |
| Model 1 | + Age Group | 954.4 | 32.25 | 0.00 | 34.25 | < 0.001 |
| Model 2 | + Face valence | 933.0 | 21.41 | 0.07 | 23.41 | < 0.001 |
| **Model 3** | **× Face valence** | **928.5** | **4.41** | **0.64** | **6.41** | **0.011** |
| Model 4 | + Touch valence | 930.5 | -1.99 | 0.24 | 0.01 | 0.917 |
| Model 5 | × Touch valence | 934.7 | -4.18 | 0.03 | 1.82 | 0.611 |
| Model 6 | + Condition | 936.1 | -1.36 | 0.01 | 0.64 | 0.424 |
| Model 7 | × Condition | 937.5 | -1.39 | 0.01 | 12.61 | 0.082 |
| Model 8 | + Gender | 939.5 | -1.99 | 0.00 | 0.007 | 0.933 |

**Table S5.** Comparison between models predicting response times. Note that smaller values of AIC indicate better fitting models.

MODEL INFO (Model 3):
Observations: 1811
Dependent Variable: RT
Type: Mixed effects generalized linear regression
Error Distribution: Gamma
Link function: inverse

MODEL FIT:
AIC = 928.54, BIC = 961.54
Pseudo-$R^2$ (fixed effects) = NA
Pseudo-$R^2$ (total) = NA

FIXED EFFECTS:

| | Est. | S.E. | t val. | p |
|---|---|---|---|---|
| (Intercept) | 0.63 | 0.06 | 10.73 | 0.00 |
| Age_Group (refrence category: Adolescent) | 0.51 | 0.08 | 6.73 | 0.00 |
| Valence.Face (ref Negative) | 0.02 | 0.01 | 3.14 | 0.00 |
| Age_Group:Valence.Face (ref Adolescent - Negative) | 0.04 | 0.02 | 2.53 | 0.01 |

ANOVA:

| | Chisq | Df | Pr(>Chisq) |
|---|---|---|---|
| (Intercept) | 115.09 | 1 | < .001 *** |
| Age_Group | 45.29 | 1 | < .001 *** |
| Valence.Face | 9.86 | 1 | 0.002 ** |
| Age_Group:Valence.Face | 6.42 | 1 | 0.011 * |

POST-HOC CONTRASTS:

---

Age_Group = Adolescent:

| model term | df1 | df2 | F.ratio | p.value |
|---|---|---|---|---|
| Valence.Face | 1 | Inf | 9.856 | 0.0017 |

Age_Group = Adult:

| model term | df1 | df2 | F.ratio | p.value |
|---|---|---|---|---|
| Valence.Face | 1 | Inf | 20.159 | <.0001 |

**Table S6.** Description of the effects predicted by the best fitting model for accuracy scores (Model 3), ANOVA and post-hoc contrasts

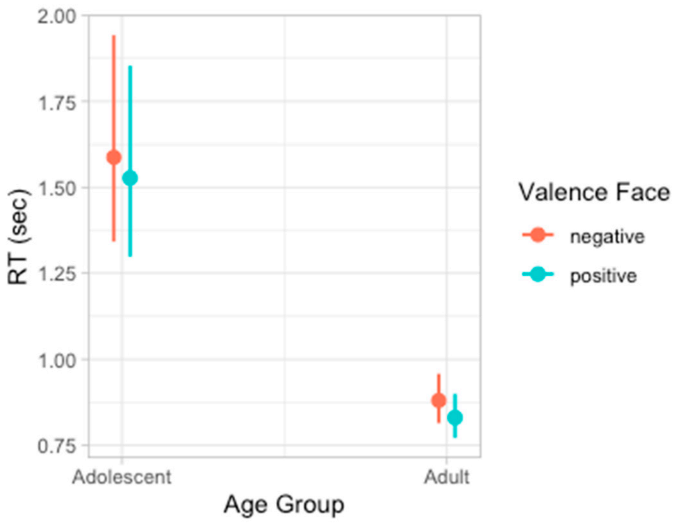


**Figure S3.** Plot of predicted values of RTs. Prediction lines and bars with confidence intervals (N participants = 60; N observations = 1811).

**Experiment 2: Accuracy**

| **Tested models** | **Variables** | **AIC** | **ΔAIC** | **wAIC** | $R^2$ | **χ2** | ***p*** |
|---|---|---|---|---|---|---|---|
| Model 0 | Random effect of Participants | 509.3 | | 0.02 | 0.35 | | |
| Model 1 | + Age Group | 506.9 | 2.43 | 0.07 | 0.34 | 4.43 | .035 |
| Model 2 | + Face valence | 507.3 | -0.39 | 0.06 | 0.34 | 1.61 | .204 |
| Model 3 | × Face valence | 509.3 | -1.99 | 0.02 | 0.34 | 0.006 | .939 |
| Model 4 | + Touch valence | 508.1 | 1.14 | 0.04 | 0.35 | 3.14 | .076 |
| **Model 5** | **× Touch valence** | **502.7** | **5.43** | **0.55** | **0.37** | **11.43** | **.010** |
| Model 6 | + Condition | 504.7 | -1.99 | 0.20 | 0.37 | 0.006 | .936 |
| Model 7 | × Condition | 508.0 | -3.24 | 0.04 | 0.83 | 10.76 | .149 |
| Model 8 | + Gender | 509.7 | -1.80 | 0.01 | 0.85 | 0.20 | .658 |

**Table S7.** Comparison between models predicting accuracy scores. Note that smaller values of AIC indicate better fitting models.

MODEL INFO (Model 5):
Observations: 1893
Dependent Variable: ACC
Type: Mixed effects generalized linear regression
Error Distribution: binomial
Link function: logit

MODEL FIT:
AIC = 502.68, BIC = 552.59
Pseudo-$R^2$ (fixed effects) = 0.09
Pseudo-$R^2$ (total) = 0.37

FIXED EFFECTS:

| | Est. | S.E. | z val. | p |
|---|---|---|---|---|
| (Intercept) | 4.18 | 0.51 | 8.19 | 0.00 |
| Age_Group (reference category Adolescent) | 0.84 | 0.80 | 1.05 | 0.29 |
| Valence.Face (ref Negative) | -1.23 | 0.47 | -2.65 | 0.01 |
| Valence.Touch (ref Negative) | -0.60 | 0.50 | -1.21 | 0.23 |
| Age_Group:Valence.Face (ref Adolescent - Negative) | 0.34 | 0.84 | 0.40 | 0.69 |
| Age_Group:Valence.Touch (ref Adolescent - Negative) | 0.30 | 0.92 | 0.33 | 0.74 |
| Valence.Face:Valence.Touch (ref Negative - Negative) | 2.18 | 0.72 | 3.01 | 0.00 |
| Age_Group:Valence.Face:Valence.Touch (ref Adolescent - Negative - Negative) | -0.99 | 1.27 | -0.78 | 0.44 |

ANOVA:

| | Chisq | Df | Pr(>Chisq) |
|---|---|---|---|
| (Intercept) | 67.07 | 1 | < .001 *** |
| Age_Group | 1.10 | 1 | 0.294 |
| Valence.Face | 7.02 | 1 | 0.008** |
| Valence.Touch | 1.46 | 1 | 0.228 |
| Age_Group:Valence.Face | 0.16 | 1 | 0.686 |
| Age_Group:Valence.Touch | 0.11 | 1 | 0.742 |
| Valence.Face:Valence.Touch | 9.06 | 1 | 0.002 ** |
| Age_Group:Valence.Face:Valence.Touch | 0.61 | 1 | 0.437 |

POST-HOC CONTRASTS:

Valence Touch = negative

| model term | df1 | df2 | F.ratio | p.value |
|---|---|---|---|---|
| Age_Group | 1 | Inf | 3.234 | 0.072 |
| Valence.Face | 1 | Inf | 6.319 | 0.012 |
| Age_Group:Valence.Face | 1 | Inf | 0.164 | 0.686 |

Valence Touch = positive

| model term | df1 | df2 | F.ratio | p.value |
|---|---|---|---|---|
| Age_Group | 1 | Inf | 1.818 | 0.178 |
| Valence.Face | 1 | Inf | 1.692 | 0.193 |
| Age_Group:Valence.Face | 1 | Inf | 0.464 | 0.496 |

**Table S8.** Description of the effects predicted by the best fitting model for accuracy scores (Model 5), ANOVA and post-hoc contrasts on the interaction effect Touch valence × Face valence

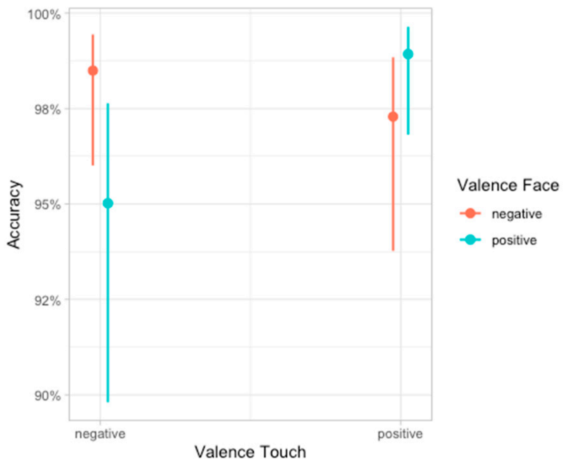


**Figure S4.** Plot of predicted values of accuracy (Experiment 2) showing the interaction between Face valence and Touch valence. Prediction lines and bars with confidence intervals (N participants = 60; N observations = 1893).

**Experiment 2: RTs**

| Tested models | Variables | AIC | ΔAIC | wAIC | χ2 | *p* |
|---|---|---|---|---|---|---|
| Model 0 | Random effect of Participants | 809.6 | | 0.00 | | |
| Model 1 | + Age Group | 792.4 | 17.20 | 0.00 | 19.20 | <.001 |
| Model 2 | + Face valence | 790.6 | 1.81 | 0.00 | 3.81 | .051 |
| Model 3 | × Face valence | 790.0 | 0.54 | 0.01 | 2.54 | .111 |
| Model 4 | + Touch valence | 788.9 | 1.08 | 0.01 | 3.08 | .079 |
| Model 5 | × Touch valence | 783.8 | 5.15 | 0.12 | 11.15 | .011 |
| Model 6 | + Condition | 779.9 | 3.89 | 0.80 | 5.89 | .015 |
| Model 7 | × Condition | 785.8 | -5.88 | 0.04 | 8.12 | .322 |
| Model 8 | + Gender | 786.4 | -0.60 | 0.03 | 1.40 | .236 |

**Table S9.** Comparison between models predicting response times. Note that smaller values of AIC indicate better fitting models.

MODEL INFO (Model 6):
Observations: 1832
Dependent Variable: RT
Type: Mixed effects generalized linear regression
Error Distribution: Gamma
Link function: inverse

MODEL FIT:
AIC = 779.88, BIC = 840.53
Pseudo-$R^2$ (fixed effects) = NA
Pseudo-$R^2$ (total) = NA

FIXED EFFECTS:

| | Est. | S.E. | t val. | p |
|---|---|---|---|---|
| (Intercept) | 0.76 | 0.08 | 9.86 | 0.00 |
| Age_Group (reference category Adolescent) | 0.44 | 0.10 | 4.47 | 0.00 |
| Valence.Face (ref Negative) | -0.02 | 0.01 | -1.71 | 0.09 |
| Valence.Touch (ref negative) | -0.01 | 0.01 | -1.37 | 0.17 |
| Condition (ref No-social) | -0.02 | 0.01 | -2.43 | 0.02 |
| Age_Group:Valence.Face (ref Adolescent - Negative) | 0.05 | 0.02 | 2.33 | 0.02 |
| Age_Group:Valence.Touch (ref Adolescent - Negative) | 0.04 | 0.02 | 1.62 | 0.11 |
| Valence.Face:Valence.Touch (ref Negative - Negative) | 0.05 | 0.02 | 3.36 | 0.00 |
| Age_Groupt:Valence.Face:Valence.Touch (ref Adolescent - Negative - Negative) | -0.06 | 0.03 | -1.65 | 0.10 |

ANOVA:

| | Chisq | Df | Pr(>Chisq) |
|---|---|---|---|
| (Intercept) | 97.22 | 1 | < 0.001*** |
| Age_Group | 20.02 | 1 | < 0.001*** |
| Valence.Face | 2.92 | 1 | 0.087 . |
| Valence.Touch | 1.89 | 1 | 0.169 |
| Condition | 5.91 | 1 | 0.015 * |
| Age_Group:Valence.Face | 5.45 | 1 | 0.020 * |
| Age_Group:Valence.Touch | 2.62 | 1 | 0.106 |
| Valence.Face:Valence.Touch | 11.28 | 1 | 0.001 *** |
| Age_Group:Valence.Face:Valence.Touch | 2.71 | 1 | 0.100 |

POST-HOC CONTRASTS:

Valence Touch = negative

| model term | df1 | df2 | F.ratio | p.value |
|---|---|---|---|---|
| Age_Group | 1 | Inf | 22.853 | <.0001 |
| Valence.Face | 1 | Inf | 0.551 | 0.4580 |
| Condition | 1 | Inf | 5.914 | 0.0150 |
| Age_Group:Valence.Face | 1 | Inf | 5.450 | 0.0196 |

Valence Touch = positive

| model term | df1 | df2 | F.ratio | p.value |
|---|---|---|---|---|
| Age_Group | 1 | Inf | 23.797 | <.0001 |
| Valence.Face | 1 | Inf | 7.719 | 0.0055 |
| Condition | 1 | Inf | 5.914 | 0.0150 |
| Age_Group:Valence.Face | 1 | Inf | 0.000 | 0.9842 |

**Table S10.** Description of the effects predicted by the best fitting model for RTs (Model 6), ANOVA and post-hoc contrasts on the interaction effect Touch valence × Face valence

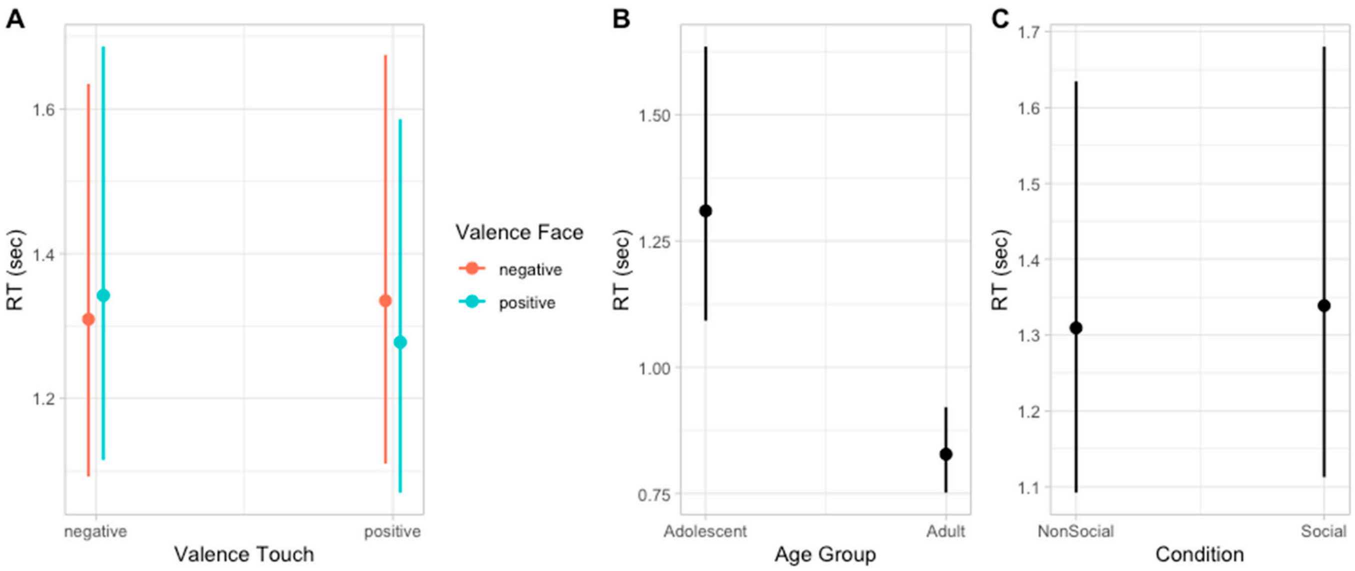


**Figure S5.** Plot of predicted values of RTs (Experiment 2) showing the interaction between Face valence and Touch valence (panel A), the main effect of Age group (panel B) and the main effect of Social condition (panel C). Prediction lines and bars with confidence intervals (N participants = 60; N observations = 1832).
